# PIWI-interacting RNAs piR-13643 and piR-21238 are promising diagnostic biomarkers of papillary thyroid carcinoma

**DOI:** 10.18632/aging.103206

**Published:** 2020-05-19

**Authors:** Zhengyan Chang, Guo Ji, Runzhi Huang, Hong Chen, Yaohui Gao, Weifeng Wang, Xuechen Sun, Jie Zhang, Jiayi Zheng, Qing Wei

**Affiliations:** 1 Department of Pathology, Shanghai Tenth People’s Hospital, Tongji University School of Medicine, Shanghai; 2 Division of Spine, Department of Orthopedics, Tongji Hospital Affiliated to Tongji University School of Medicine, Shanghai, China; 3 Key Laboratory of Spine and Spinal Cord Injury Repair and Regeneration (Tongji University), Ministry of Education, Shanghai, China; 4 Center for Difficult and Complicated Abdominal Surgery, Shanghai Tenth People’s Hospital, Tongji University School of Medicine, Shanghai, China; 5 Central Laboratory, Shanghai Tenth People's Hospital, Shanghai, China; 6 Department of Prevention, Tongji University School of Medicine, Tongji University, Shanghai, China; 7 Human Province Key Laboratory of Tumor Cellular and Molecular Pathology, Cancer Research Institute, University of South China, Hengyang, China

**Keywords:** PIWI-interacting RNAs, thyroid cancer, biomarkers, diagnosis, nomogram

## Abstract

Emerging studies demonstrate that PIWI-interacting RNAs (piRNAs) participate in the development of cancers. 75 pairs of papillary thyroid carcinoma (PTC) samples and 31 benign thyroid nodule samples were included in this three-phase biomarker identifying study. First, piRNA expression profiles of five pairs of PTC samples were acquired piRNA sequencing. The expression of all upregulated piRNAs were further validated by RT-qPCR. Paired t and nonparametric test were used to evaluate the association between all upregulated piRNAs and clinic stage. The expression levels of key piRNAs were corrected by demographic data to construct a multivariate model to distinguish malignant nodules from benign. Additionally, the intersection between target genes of key piRNAs and differentially expressed genes in The Cancer Genome Atlas (TCGA) PTC samples were used to perform enrichment analysis. Only piR-13643 and piR-21238 were significantly upregulated in PTC and associated with clinic stage. Moreover, both piR-13643 (Area Under Curve (AUC): 0.821) and piR-21238 (AUC: 0.823) showed better performance in distinguishing malignant nodules from benign than currently used biomarkers HBME1 (AUC: 0.590). Based on our findings, piR-13643 and piR-21238 were observed to be significantly upregulated in human PTC. PIWI-interacting RNAs could serve as promising novel biomarkers for accurate detection of PTC.

## INTRODUCTION

Papillary thyroid carcinoma (PTC) is identified as the dominating histological subtype of thyroid cancer, approximately accounting for 87% of the thyroid malignant diagnosed in the US [1]. The incidence of new thyroid cancer has increased by an average rate of 3.1% each year over the last decade. PTC metastasizes primarily through the lymph nodes, which increases the risk of postoperative recurrence [2]. Currently, in the field of early detection of PTC, imaging and ultrasound are the predominant screening methods. Although these methods are helpful for the detection of PTC, the screening effectiveness sometimes is limited by the depth and size of the lesion [3, 4]. Thus, it is essential to develop a simple and effective tissue-based test that improves diagnostic rates for PTC. Recently, various biomolecules including proteins, DNA, mRNAs, and miRNAs have shown great potential in several previous studies to serve as new biomarkers for the prognosis prediction, and diagnosis of PTC [5–7]. Novel biomarkers based on these molecules may be suitable for diagnosis and monitoring disease progression of PTC.

Numerous studies have discovered a category of non-coding RNAs called P-element induced Wimpy testis (PIWI)-interacting RNAs (piRNAs) that are abundant in various types of tissues [8, 9]. PiRNAs are a class of small non-coding RNAs of 26–31 nucleotides in length interacting with PIWI proteins, which can post-transcriptionally and epigenetically silence the transposable elements in germline stem cells [10–13]. Besides, the expression levels of piRNAs have been reported to associate with the tumorgenesis and progression of several types of cancers [14–16]. Because of their small size, piRNAs are not readily degraded by ribonucleases and can allodially pass through cell membranes [17]. Although changes in piRNA levels have recently been linked to human diseases, their roles and functions in malignancy remain unclear. Investigations of the possible clinical relevance and biological functions of piRNAs in PTC are still in early stages.

PTC is a relatively indolent cancer based on investigations using imaging and fine-needle aspiration (FNA) biopsy. Although PTC patients often have good prognosis, some patients still die of tumor recurrence and metastasis. Therefore, early diagnosis and prediction of tumor metastasis are crucial to the prognosis of some patients. We have found that some PTC patients did not have typical morphological characteristics and molecular biological phenotypes, such as BRAF mutations. The purpose of this study is to find biomarkers that could be used in combination with traditional biomarkers, such as BRAF mutations and immunohistochemical staining of HBME1, to optimize the early diagnosis of PTC.

To the best of knowledge, this study compared the expression of piRNAs in PTC and normal thyroid tissues by Next Generation Sequencing (NGS) in concert with subsequent Reverse Transcription Quantitative Polymerase Chain Reaction (RT-qPCR) validation firstly. The aim of this study was to identify piRNAs with abnormal expression in PTC, which also had diagnostic or differential diagnostic value and could potentially serve as biomarkers for early detection of PTC. The results of this study might provide novel biomarkers for the accurate detection of PTC.

## RESULTS

### Differential expression analysis

**Figure 1:**
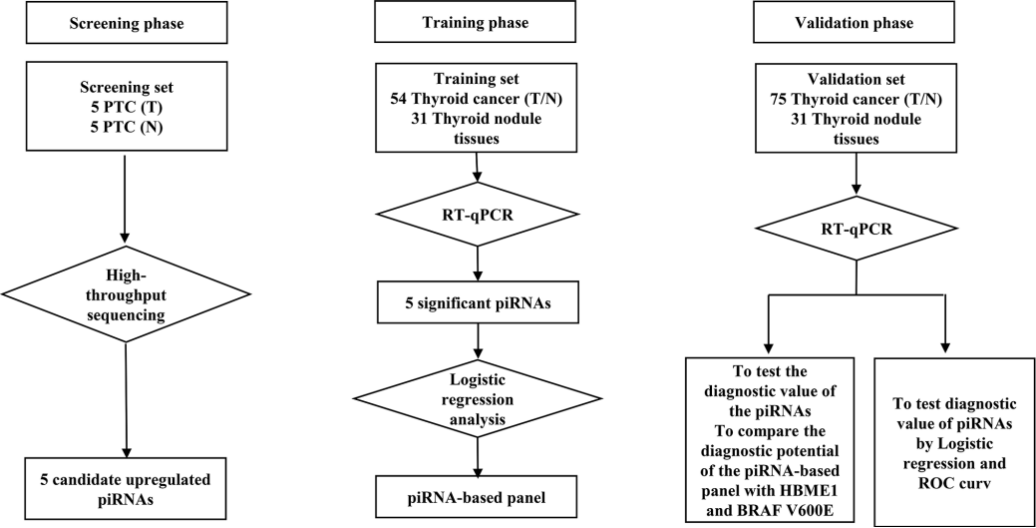
**Flow diagram of the study design illustrating how the patients and controls were divided into screening, training and validation phase of the study.**

**Figure 2:**
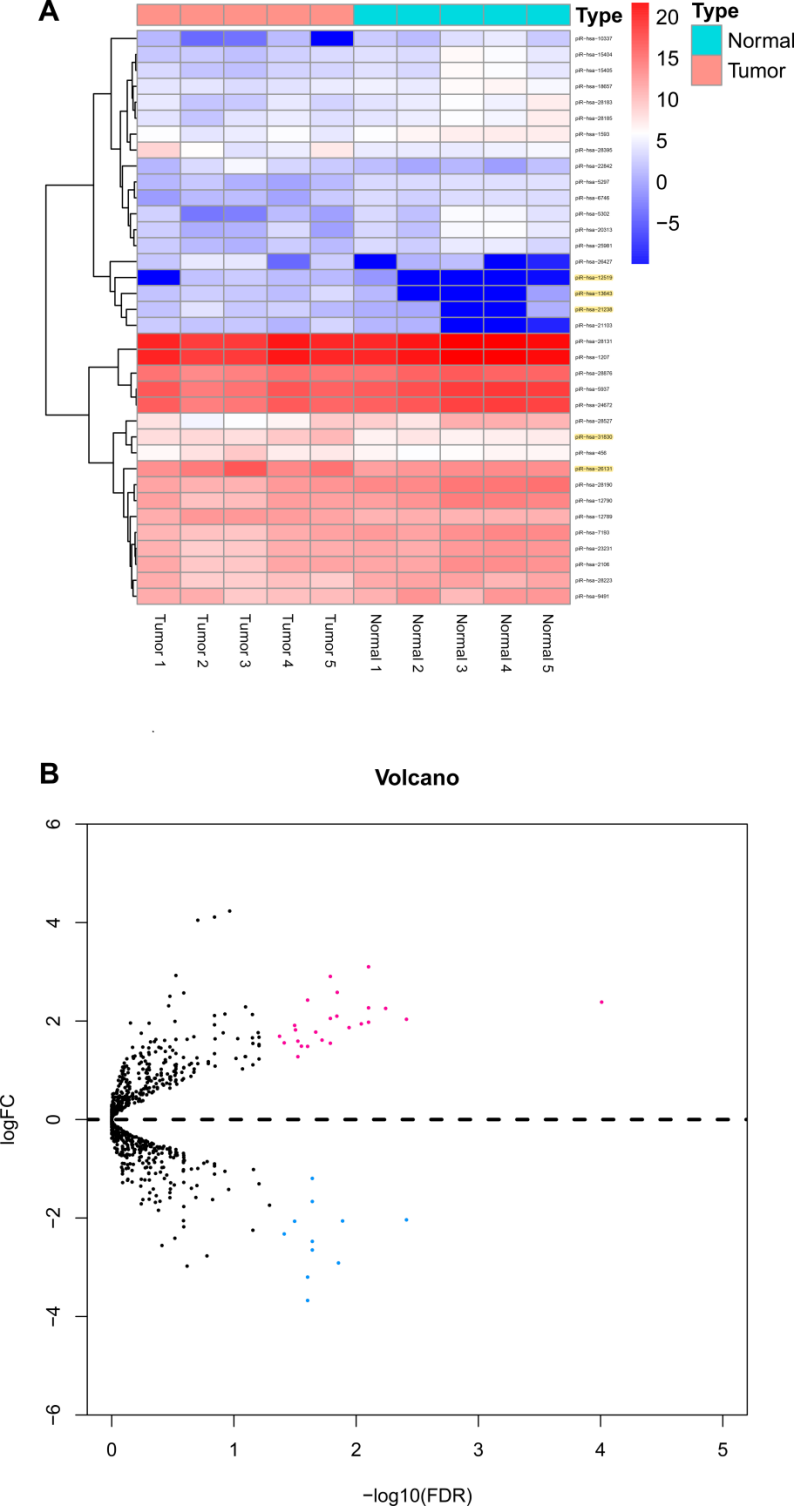
**Differential piRNAs changes in papillary thyroid carcinoma tissues compared with normal thyroid.** (**A**) Result of hierarchical clustering for top 35 differential piRNAs. (**B**) Result of volcano for five pairs of papillary thyroid carcinoma tissues.

**Figure 3:**
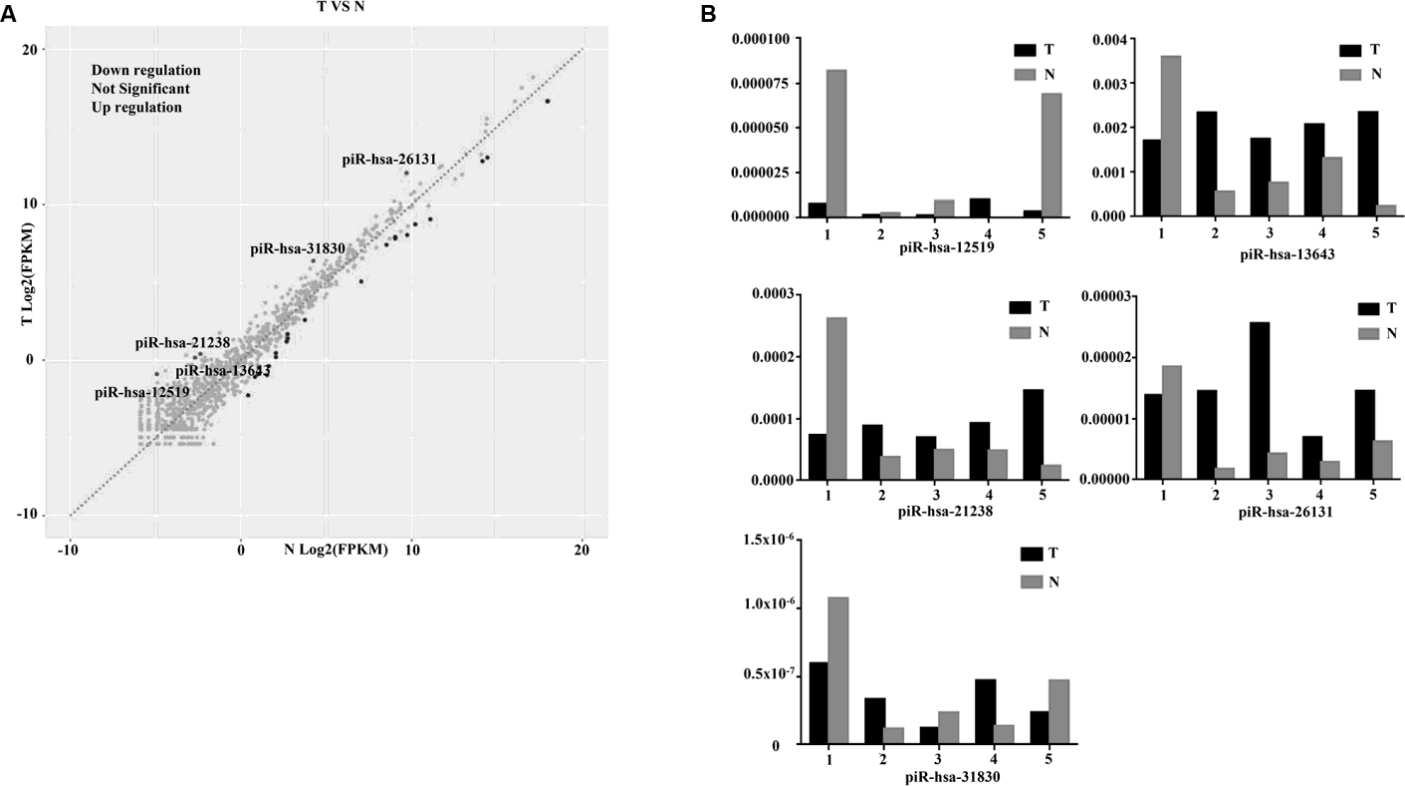
**Diagnostic performance of piR-13643 and piR-21238 in the screening phase of the study.** (**A**) The scatter plot was used for assessing the piRNAs expression variation. (**B**) Based on the results of NGS, five piRNAs (piR-31830, piR-13643, piR-26131, piR-12519, piR-21238) were chosen as potential endogenous controls for normalization of RT-qPCR data.

The three-phase study design is described in Figure 1. Clinical and pathological characteristics of the PTC tissue samples are summarized in Table 1. In total, ten small RNA libraries of five pairs of PTC and normal solid tissue were prepared and sequenced. Compared to normal tissues, the expression of 35 piRNAs were found to be significantly abnormal in PTC tissues (Figures 2A, 2B, and 3A). Based on the selection criteria of differential expression genes (DEGs) described in the methods, five piRNAs were chosen for further RT-qPCR evaluation (Table 2). Among these five, piRNA-26131, piR-13643 and piR-21238 were also differentially expressed in RT-qPCR data and selected for the further analysis (Figure 3B).

**Table 1:** Clinicopathological characteristics of study subjects.

**Clinical characteristic**	**Screening phase**	**Training phase**	**Validation phase**
Number	5	54	21
Age (years)			
< 45	3	29	10
≥ 45	2	25	11
Gender			
Male	2	19	3
Female	3	35	18
TNM stage			
Stage I	4	33	15
Stage II	0	3	4
Stage III	1	9	0
Stage IV	0	9	2
Microcarcinoma			
Yes	0	5	9
No	5	49	12
Hashimoto's thyroiditis			
Yes	3	16	11
No	2	30	10
Unkonwn	0	8	0
Multifocal			
Yes	1	4	3
No	4	50	18
LN metastasis			
Yes	4	38	6
No	1	16	15
HMBE1			
-	1	26	11
+	3	13	6
++	1	15	4
BRAF			
WT	2	13	21
V600E	3	41	0
Recurrence			
Yes	4	18	6
No	1	36	15
Metastasis			
Yes	3	6	3
No	2	48	18

**Table 2:** The list of piRNAs significantly upregulated in tumor tissue of papillary thyroid carcinoma compared to normal tissue by next-generation sequencing.

**Up-regulated piRNA**	**Fold change**	**FDR**
piR-hsa-31830	4.409006161	5.77E-05
piR-hsa-13643	7.228884305	0.000239
piR-hsa-26131	5.03247354	0.000455
piR-hsa-12519	16.39352629	0.00082
piR-hsa-21238	6.841397067	0.000849

Abbreviation: PIWI-interacting RNAs (piRNAs); Fold Change (FC).

### Quantification of upregulated piRNAs by RT-qPCR

**Figure 4:**
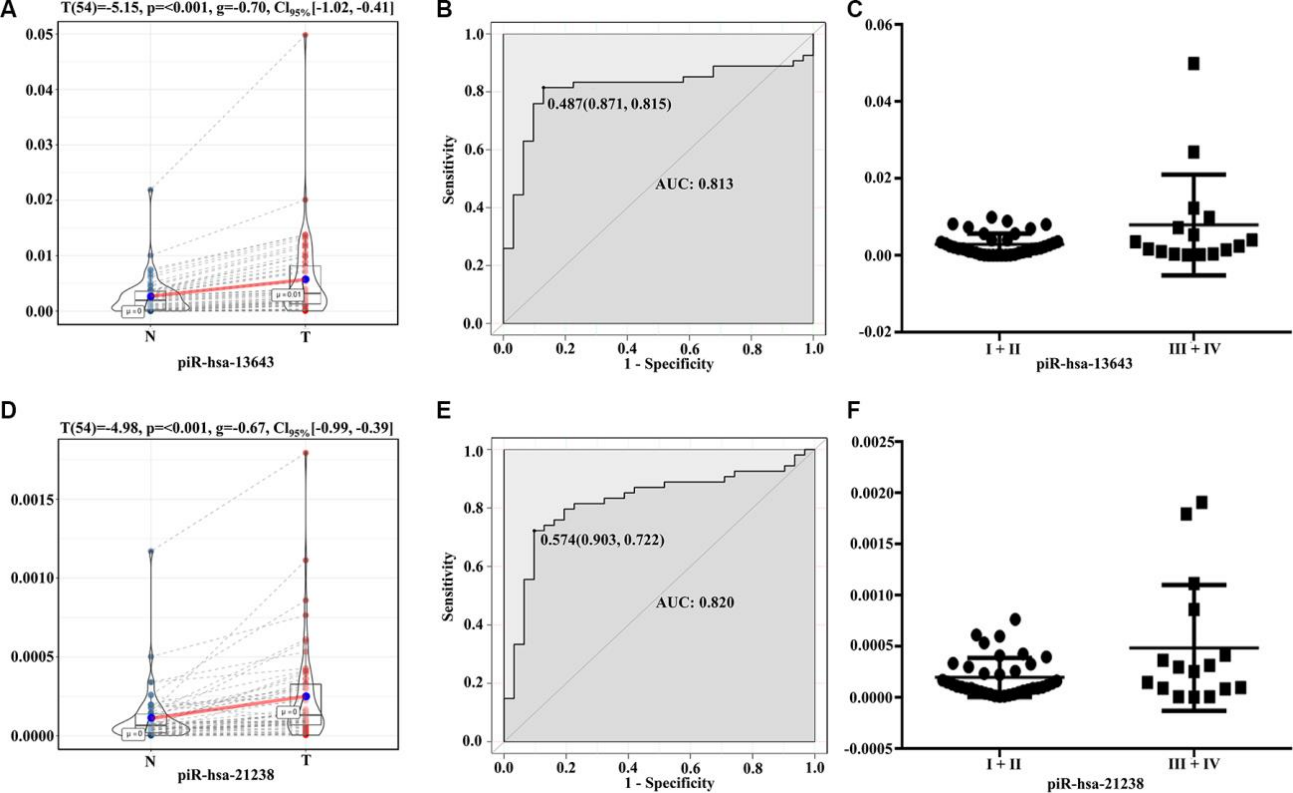
**Diagnostic performance of piR-13643 and piR-21238 in the training phase of the study.** (**A**) The expression of piR-13643 is significantly upregulated in tumor tissue of papillary thyroid carcinoma patients compared to normal tissue (P < 0.001). (**B**) ROC analyses based on the expression of piR-13643 (AUC = 0.813). (**C**) The levels of piR-13643 increase significantly with advanced clinical stage (P = 0.0258). (**D**) The expression of piR-21238 is significantly upregulated in tumor tissue of papillary thyroid carcinoma patients compared to normal tissue(P < 0.001). (**E**) ROC analyses based on the expression of piR-21238 (AUC = 0.820). (**F**) The levels of piR-21238 decrease significantly with advanced clinical stage (P =0.0112). *P < 0.05; **P < 0.01; ***P < 0.001

**Figure 5:**
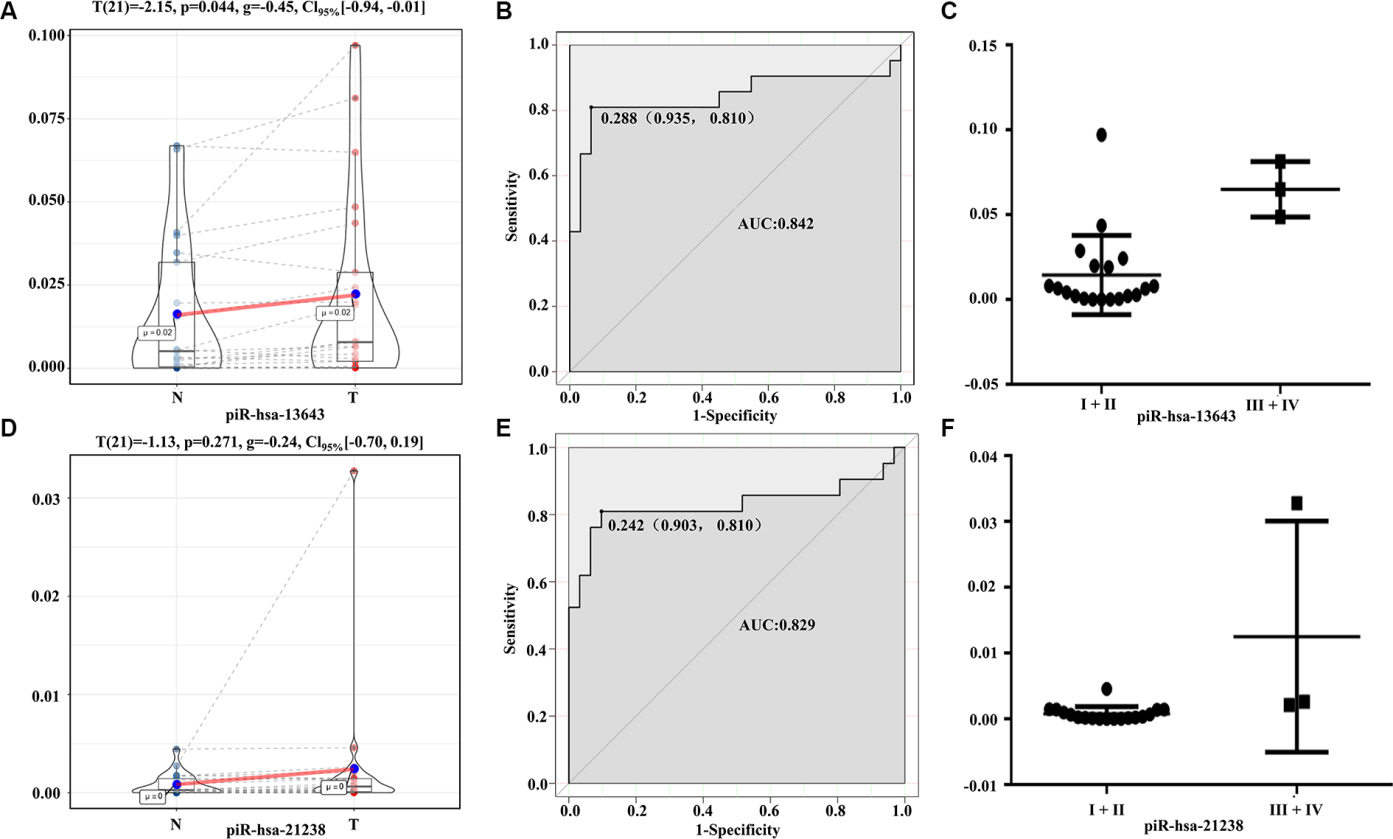
**Diagnostic performance of piR-13643 and piR-21238 in the validation phase of the study.** (**A**) The expression of piR-13643 is significantly down-regulated in tumor tissue of papillary thyroid carcinoma patients compared to normal tissue (P=0.044). (**B**) ROC analyses based on the expression of piR-13643 (AUC = 0.842). (**C**) The levels of piR-13643 increased significantly with advanced clinical stage (P = 0.0019). (**D**) The expression of piR-21238 is significantly upregulated in tumor tissue of papillary thyroid carcinoma patients compared to normal tissue (P =0.271). (**E**) ROC analyses based on the expression of piR-21238 (AUC = 0.829). (**F**) The levels of piR-21238 increased significantly with advanced clinical stage (P =0.0042). *P < 0.05; **P < 0.01; ***P < 0.001

The expression levels of piR-13643 (P < 0.001, Figure 4A; P = 0.044, Figure 5A) and piR-21238 (P < 0.001, Figure 4D) (P = 0.271, Figure 5D) were higher in PTC tissues than in non-cancerous tissue controls in both fresh and paraffin-embedded samples. In addition, the receiver operating characteristic (ROC) curves indicated good discrimination of piR-13643 (AUC = 0.813, Figure 4B) (AUC = 0.842, Figure 5B) and piR-21238 (AUC = 0.820, Figure 4E) (AUC = 0.829, Figure 5E) from controls in the diagnosis of PTC. Furthermore, the expression of piR-13643 (P = 0.026, Figure 4C; P = 0.002, Figure 5C) and piR-21238 (P = 0.011, Figure 4F; P = 0.004, Figure 5F) increased significantly in samples of PTC at advanced clinical stages (Table 3).

**Table 3:** Two-phase validation of upregulated piRNAs identified by real-time PCR.

**Up-regulated piRNA**	**Fold change**	**FDR**	**AUC**
54 paired fresh samples			
piR-hsa-13643	3.588852	0.0002	0.813
piR-hsa-21238	3.537825	0.0004	0.820
21 paired paraffin samples			
piR-hsa-13643	3.462167	0.0434	0.842
piR-hsa-21238	5.437552	0.0453	0.829

Abbreviation: PIWI-interacting RNAs (piRNAs); Fold Change (FC); Area Under Curve (AUC).

### Construction of nomogram distinguishing malignant nodules from benign ones

**Figure 6:**
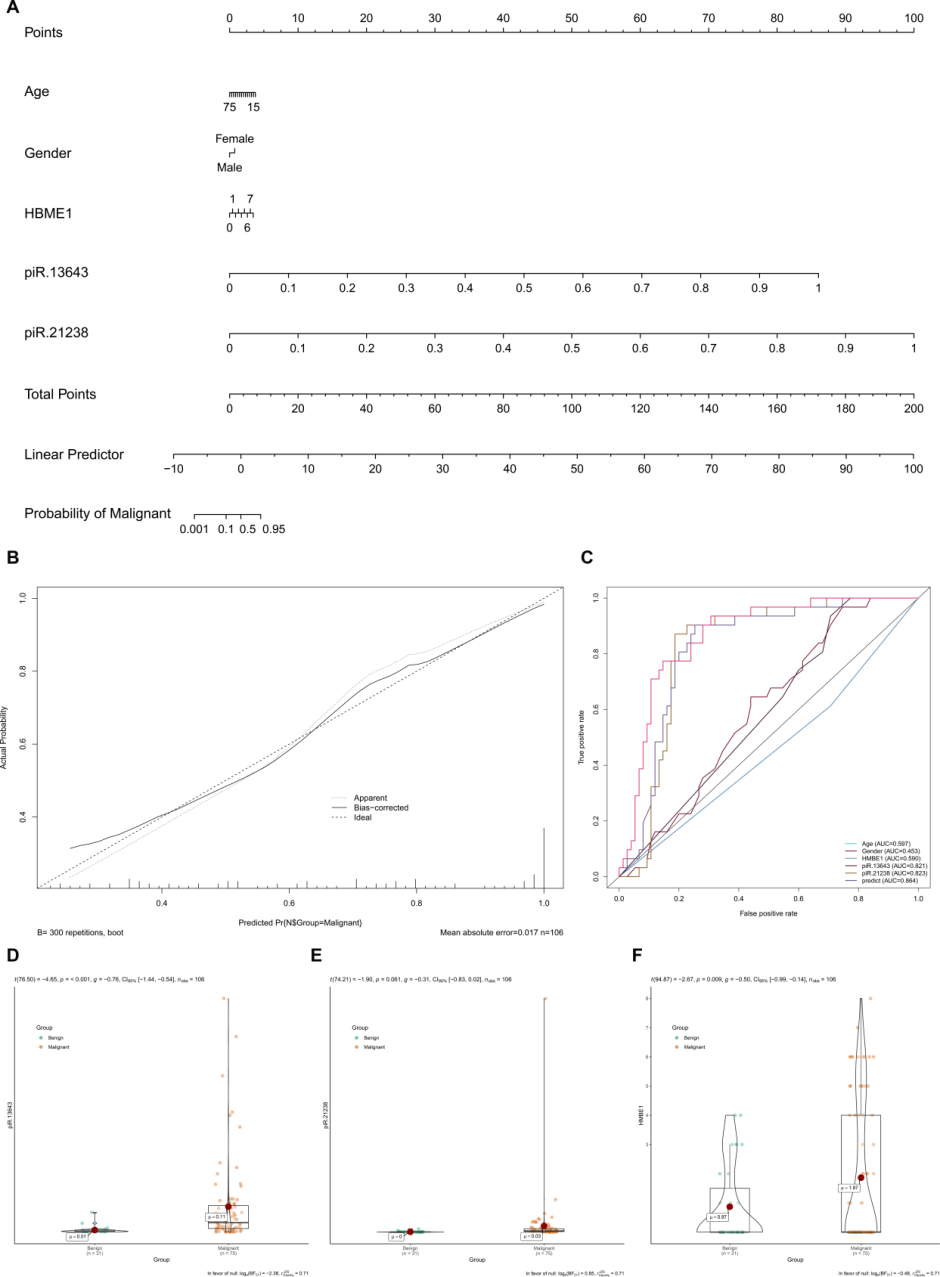
**The expression levels of key piRNAs were corrected by demographic data to construct a multivariate model to distinguish malignant nodules from benign.** (**A**) Nomogram based on multivariate model (**A**). The calibration curve and ROC curves suggested acceptable calibration (**B**) and discrimination (**C**) of the nomogram, respectively. Additionally, piR-13643 (P < 0.001), piR-21238 (P = 0.061) and HBME1 (P = 0.009) were higher in malignant nodules compared to benign nodules in both fresh and paraffin samples (**D**–**F**).

HBME1 staining and BRAF^V600E^ mutations were detected in less than 50% of the PTC samples, while the upregulation of piR-13643 and piR-21238 was detected in 62% of all PTC samples (Table 1 and Supplementary Figure 1 and Supplementary Material 1). The expression levels of these two key piRNAs were corrected by demographic data to construct a multivariate model to distinguish malignant from benign nodules (Figure 6A). The calibration and ROC curves indicated acceptable data calibration and discrimination of the nomogram, respectively (Figure 6B, 6C). Both piR-13643 (AUC: 0.821) and piR-21238 (AUC: 0.823) showed better performance in distinguishing malignant from benign nodules than HBME1 staining alone (AUC: 0.590). Expression of piR-13643 (P < 0.001) and piR-21238 (P = 0.061), and staining of HBME1 (P = 0.009) were higher in PTC nodules compared with benign nodules in both fresh and paraffin-embedded samples (Figure 6D–6F). In some patients, we found examples of HBME1 staining in benign nodules and non-staining in PTC nodules (Supplementary Figure 2).

### Identification of key piRNAs potential downstream mechanisms

The RNAhybrid algorithm determined that there were 2,711 and 11,192 potential target genes of piR-13643 and piR-21238, respectively [51]. FASTA sequence files of piR-13643 (https://www.ncbi.nlm.nih.gov/nucleotide/DQ583334) and piR-21238 (https://www.ncbi.nlm.nih.gov/nucleotide/DQ590959) were obtained from the NCBI Nucleotide database. The target binding sites of piR-13643 and piR-21238 are presented in Supplementary Material 2.

Based on the data from The Cancer Genome Atlas Thyroid Cancer project (TCGA-THCA), 5,555 DEGs in PTC tissues were identified (Figure 7A, 7B). Between primary PTC with and without metastasis, 468 DEGs were identified (Figure 7C, 7D). Of all the DEGs identified, 306 were potential target genes of piR-13643 and 1,434 were potential target genes of piR-21238 (Figure 7E–7H). Functional enrichment analysis suggested that some important gene ontologies and pathways such as “skeletal system development”, “extracellular structure organization”, “PI3K−Akt signaling pathway”, and “MAPK signaling pathway” might be potential downstream targets of piR-13643 and piR-21238. A mechanism diagram of the most reported piRNA downstream targets in cancer (Figure 8) was obtained from a review of the literature [17–21].

**Figure 7:**
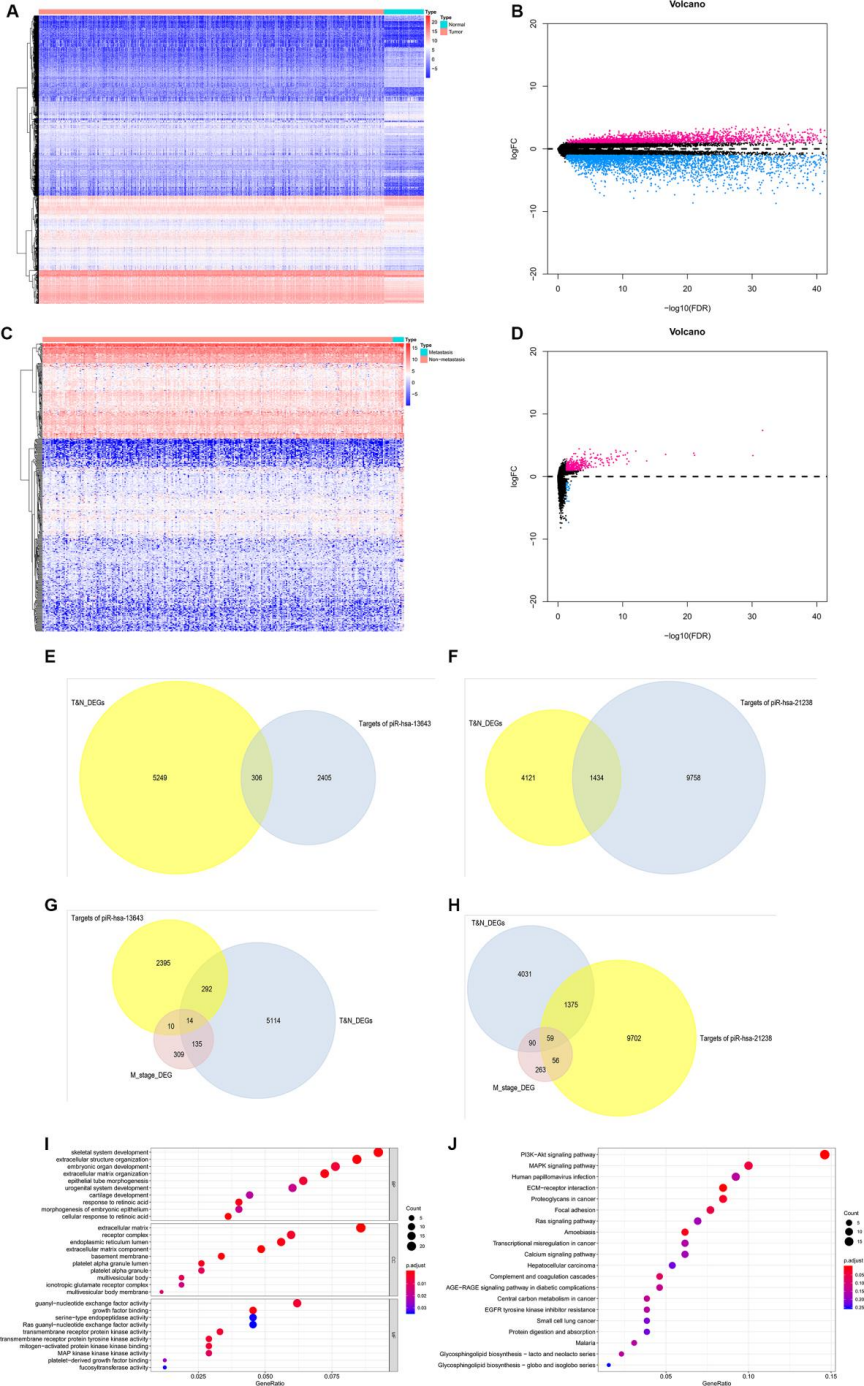
**Validation of target genes of piRNA-13643 and piRNA-21238.** (**A**) Result of hierarchical clustering for differential genes in thyroid cancer and noncancerous tissue was calculated by TCGA database. (**B**) Result of volcano for differential genes in thyroid cancer and noncancerous tissue was calculated by TCGA database. (**C**) Result of hierarchical clustering for differential genes in thyroid cancer with or without metastasis tissue was calculated by TCGA database. (**D**) Result of volcano for differential genes in thyroid cancer with or without metastasis tissue was calculated by TCGA database. (**E**) piRNA-13643 target genes prediction using differential genes in thyroid cancer and noncancerous tissue in TCGA database. (**F**) piRNA-21238 target genes prediction using differential genes in thyroid cancer and noncancerous tissue in TCGA database. (**G**) piRNA-13643 target prediction using differential genes in thyroid cancer with or without metastasis tissue in TCGA database. (**H**) piRNA-21238 target prediction using differential genes in thyroid cancer with or without metastasis tissue in TCGA database. (**I**) Gene Ontology (GO) analysis of piRNA-13643 associated with biological process, molecular function and cellular component. (**J**) Top twenty signal pathways of piRNA-13643 by KEGG.

**Figure 7:**
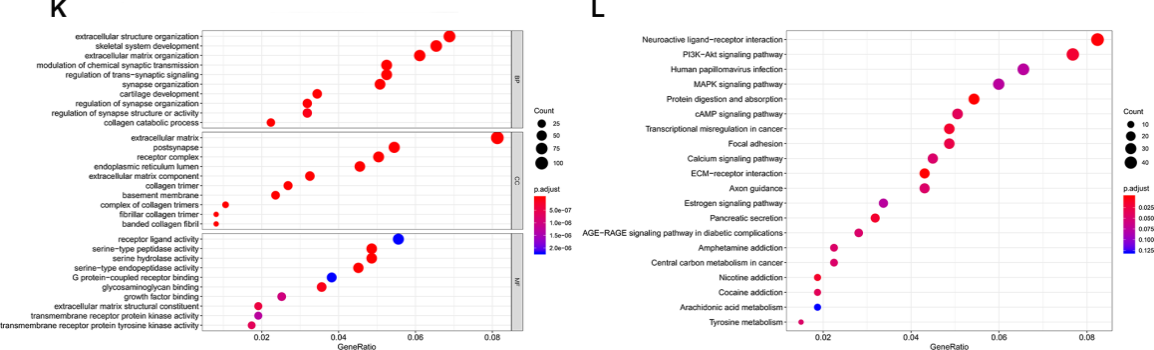
**Validation of target genes of piRNA-13643 and piRNA-21238.** (**K**) Gene Ontology (GO) analysis of piRNA-21238 associated with biological process, molecular function and cellular component. (**L**) Top twenty signal pathways of piRNA-21238 by KEGG.

**Figure 8:**
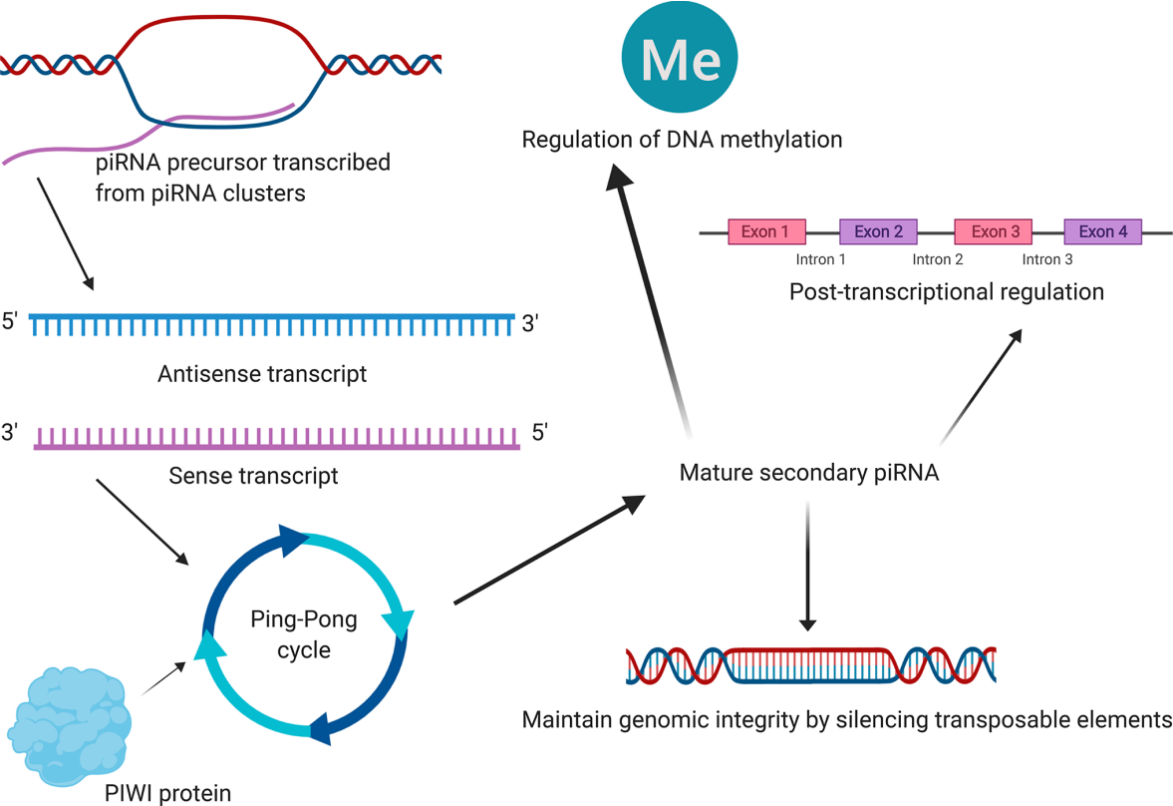
**The mechanism diagram (schematic diagram) of the most reported piRNA downstream mechanisms in cancer obtained from literature reviewing [17–21].** Emerging studies revealed that piRNAs were associated with the development and progression of several types of cancers, primarily through maintaining genomic integrity by silencing transposable elements, post-transcriptional regulation and regulation of DNA methylation. Although this summary might not be comprehensive, they were by far the most reported piRNA downstream mechanisms in cancer. And Figure 8 illustrates a mechanism diagram of the most reported piRNA downstream mechanisms in cancer obtained from literature reviewing.

## DISCUSSION

The data from the Epidemiology, Surveillance, and End Results (SEER) indicate that about 1.2% of the population will be diagnosed with thyroid cancer during their lifetime in recent years [22]. By an average incidence of 3.1% and death rates of 0.7% each year, the number of thyroid cancer patient has increased over the last decade [2, 23, 24]. As the aging population degree deepens, there will be more and more elderly PTC patients with long-term survival.

Despite such a dramatic rise in incidence and mortality of PTC has remained stable in recent years, there are more and more widespread over-diagnosis cases that is not destined to cause death or clinical illness [25–27]. In clinical practice, most of these cases are due to the lack of effective indicators for early diagnosis of PTC and differential diagnosis of benign and malignant thyroid nodules. Besides, some patients with thyroid nodules are excessive anxiety, further causing excessive treatment, which also greatly increase the medical expenses for maintaining thyroid function. And the dysfunction caused by surgical complications result in the burden of the family and society. Consequently, efforts to reduce thyroid cancer mis-detection and misdiagnosis are clearly warranted [27, 28].

In terms of pathological diagnosis, FNA is a critical tool in the diagnosis of thyroid malignancy. As the tumor progression to terminal stage, it develops invasive growth, capsular/vascular invasion and metastasis, which has typical morphological features [29]. And diagnosis is not difficult at this stage. However, the more advanced the tumor, the fewer opportunities and treatments there are for the patients. Because the diagnosis of PTC relies heavily on morphology, diagnosing PTC can be particularly challenging in early stage specimens with low cellularity [30].

Since thyroid cancer, especially PTC, has attracted increasing attention in tumor prevention and treatment in recent years, more and more studies have focused on specific diagnostic biomarkers for PTC. For example, galectin-3 has been reported to have more than 70% sensitivity and more than 90% specificity in diagnosis of PTC. Although galectin-3 has a great application prospect, it cannot be widely used in clinical practice due to the limitation of its detection technology [31, 32]. In addition, BRAF ^V600E^ gene mutation is significantly associated with PTC recurrence and prognosis. A search of the TCGA-THCA database identified and characterized > 96% of driver mutations of PTC, finding BRAF (mutation in 59.7% PTC), NRAS (mutation in 8.5% PTC) and HRAS (mutation in 3.5% PTC) as the most common causative genes [33, 34]. Our data are consistent with previous studies confirming that BRAF^V600E^ gene mutation is important in the diagnosis of PTC [35–37]. However, BRAF also has been reported to mutate in benign thyroid nodules and is not suitable for early diagnosis of thyroid cancer [30, 38, 39]. We have also found that, in clinical practice, nearly half of surgically defined PTC patients do not have BRAF ^V600E^mutations. Therefore, there are still no effective biomarker for early diagnosis of PTC and distinguishing benign and malignant thyroid nodules [30].

Growing evidences have demonstrated the critical regulatory role of piRNAs in the epigenetics of cancers [40–42]. And several studies have shown that some piRNAs are promising diagnostic biomarkers of human malignance such as colon cancer, breast cancer and lung cancer [42–45]. Additionally, piRNAs are extremely stable and not easy to degradation by ribonucleases and may play an important role in cellular communication and other biological processes. PiRNA stability may contribute to their use as potential biomarkers for malignant diseases. Yang et al. have reported that piR-57125 can be serve as a diagnostic biomarker for colon cancer for its good diagnosis performance, resistance to repeated freeze-thaw cycle and long-term room temperature incubation [8]. Additionally, the results of this study revealed that elevated levels of HBME1 staining and BRAF^V600E^ mutations were detected in less than 50% of PTC patients, while the abnormal upregulation of piR-13643 and piR-21238 were detected in 62% of all samples. However, we found that the highest diagnosis accuracy was achieved when a combination of all three biomarkers were used in the diagnosis of PTC. Thus, we concluded that piRNAs could serve as biomarker for the diagnosis of PTC and distinguishing benign and malignant thyroid nodules. But their involvement in carcinogenesis needs to be further established.

In terms of regulatory mechanisms, recent studies have demonstrated that piRNAs are significantly related to the tumorigenesis and progression of several types of cancers. This occurs primarily through maintaining genomic integrity (by silencing transposable elements), regulating post-transcriptional processes, and regulating DNA methylation [17–21]. PIWI proteins, including PIWIL1, PIWIL2, PIWIL3, and PIWIL4, have also been shown to play critical roles in tumorigenesis, progression, and poor prognosis [18, 46–49]. The results of the functional enrichment analysis showed that the most enriched GO terms included biological process, molecular function, and cellular component processes. KEGG signaling pathway enrichment analysis suggested that MAPK signaling pathway, PI3K-Akt signaling pathway and interferon response pathway have the most biological significance in the downstream mechanism of key piRNAs.

On one hand, it has been reported that signaling through the PI3K-Akt signaling pathway is often abnormally deregulated in human malignance. Deregulation of PI3K-Akt signaling pathway promotes cell survival/proliferation and immune infiltration. Several previous studies have demonstrated that PI3K-Akt pathway can be a target for treatment in some tumors [50, 51]. On the other hand, the MAPK signaling pathway employs a series of protein kinases that transmit signals from the cell membrane to the nucleus to control cellular processes such as proliferation, differentiation, apoptosis, migration, and invasion. MAPK hyperactivation has been implicated in many different pathologies [51]. Primarily through activation of MAPK and PI3K signaling cascades, genetic alternations have been reported to be prominently responsible for the onset, tumorigenesis, progression, and dedifferentiation in several types of cancer [52]. In the future, the relationship between these pathways and piRNAs in the PTC pathogenesis needs further research.

There are potential limitations in this study that should be addressed to construct piRNAs as novel diagnostic tools for PTC. First, the quantity of the free-circulating RNAs in PTC tissue can vary from patient to patient, biasing the results. Kits for small non-coding RNA library preparation are available that efficiently tag piRNAs of low abundance, which can solve this problem. Second, although the protocols of PTC sample collection and RT-qPCR was the same throughout the study, tested samples were stored for different lengths of time. This may have contributed to our observation of a small shift in piRNA expression. Finally, the relatively small sample size may impair the transformation and confidence of the models in other independent datasets.

To establish final cut-off values for piR-13643 and piR-21238, prospective validation of our model in multicenter studies need to be carried out. Our data underscore the enormous potential for the use of novel piRNA biomarkers in combination with other biomarkers for the early diagnosis of PTC. Although their regulation mechanisms and clinical significance need further evaluation, piRNA involvement in PTC pathogenesis is evident.

## CONCLUSIONS

Our investigations demonstrated that piR-13643 and piR-21238 are abundant and upregulated in PTC. In combination with the use of other biomarkers, detecting the upregulation of these two piRNAs improves the diagnosis rate of PTC. Although piRNAs could serve as promising diagnosis biomarkers for the accurate detection of PTC, their involvement in specific carcinogenetic pathways needs to be investigated.

## MATERIALS AND METHODS

### Patient selection and data extraction

PTC tissue samples in the study were harvested from the Shanghai Tenth People’s Hospital between November 2016 and August 2019. These patients were further divided into screening, training, and validation sets based on their TNM stage. Five pairs of PTC tissues were included in the screening phase, fifty-four pairs of PTC tissues were included in the training phase, and twenty-one pairs of PTC FFPE tissues were analyzed in the validation phase of this study. Information on gender, age, Union for International Cancer Control (UICC) TNM stage, and American Joint Committee on Cancer (AJCC) Cancer Staging System of each patient was collected. Patients with chronic diseases (hypertension, diabetes, coronary heart disease, etc.), other primary tumors and metastatic tumors, and patients with reliable baseline information were excluded. This study was approved by the Clinical Research Ethics Committee of Shanghai Tenth People’s Hospital. Written, informed consents were obtained from all participants in the study.

### RNA extraction

Total RNA enriched for piRNA was isolated from PTC tissues using the Qiagen miRNeasy Mini Kit (catalog #217004, Qiagen, Germany) according to the protocol of the manufacturer with modifications. Briefly, 700 μl of QIAzol lysis reagent was added to each sample and the sample was homogenized at room temperature for 5 min. Each sample received 140 μl of chloroform and was centrifuged for 15 min at 12,000 g and 4 °C. The upper aqueous phase of each sample was transferred to a new collection tube and 525 μl of 100% ethanol was added. The samples were centrifuged at 8,000 g for 15 s at room temperature. For each sample, 700 μl of RWT buffer was added to the RNeasy Mini column and centrifuged 15 s at 8,000 g. Samples of 500 μl in RPE buffer were loaded onto the RNeasy Mini column and centrifuged 15s at 8,000 g. Elution of piRNAs was performed with two 15 ul volumes of preheated Elution Solution (total volume = 30 μl).

Total RNA enriched for small RNAs was isolated from FFPE tissues using the Qiagen miRNeasy Mini Kit (catalog #217504, Qiagen, Germany) according to the protocol of the manufacturer with modifications. Briefly, 5-20 um sample sections were placed into a collection vessel containing an appropriate volume of deparaffinization solution and incubated at 56 °C for 3 min. Each sample received 150 μl PKD Buffer and 10 μl proteinase K and incubated at 56 °C for 15 min, then at 80 °C for 15 min. The sediment was transferred into a new 2 ml microcentrifuge tube and incubated on ice for 3 min. The samples were centrifuged for 20 min at 20,000 g. The supernatants were transferred to a new microcentrifuge tube and 10 μl Dnase I, 320 μl RBC buffer, and 1,120 μl ethanol were added to the sample, and 700 μl of the sample was transferred to a Rneasy MinElute spin column placed in a 2ml collection tube. The spin column was centrifuged for 15 s at 8,000 g. For each sample, 500 μl of RPE buffer was added to the Rneasy MinElute spin column and centrifuged for 15 s at 8,000 g to wash the spin column membrane. PiRNAs were eluted with two 15 μl volumes of Elution Solution. The concentration and purity of RNA were determined by measuring its optical density (A260/280 > 2.0; A260/230 > 1.8) by a NanoDrop ND-1000 Spectrophotometer (Thermo Fisher Scientific, Wilmington, DE, USA).

### Construction and sequencing of small RNA library

Ten small RNA libraries were sequenced, each containing pooled RNA from five pairs of PTC and para-cancer tissues. Total RNA was extracted using the miRNeasy Mini Kit (Cat # 217004, Qiagen) following the manufacturer’s instructions. An RIN number was determined to check RNA integrity using an Agilent Bioanalyzer 2100 (Agilent Technologies, US). Equimolar amounts of each library were pooled for a final concentration of 2 μg cDNA, and samples sequenced with 50bp single-end reads using a MiSeq sequencer (Illumina, USA). The sequencing process was controlled by the data collection software provided by Illumina for real-time data analysis (Shanghai biotechnology cooperation).

### Quantification of upregulated piRNAs by RT-qPCR

One μg of total RNA was reverse-transcribed into cDNA by a Bulge-Loop piRNA-specific RT primer and reverse transcriptase (Ribobio, Guangzhou, Guangdong, China). Reaction mixtures were incubated for 60 min at 42 °C, 10 min at 70 °C and then held at 4 °C. From the resulting reaction, 2 μl was used for quantitative PCR analysis performed on an ABI PRISM 7900 Sequence Detection System (Applied Biosystems, USA) using the miScript SYBR Green PCR Kit and Bulge-Loop primer (Ribobio, Guangzhou, Guangdong, China). A previously published method was used for piRNA detection [53, 54]. For the quantitative PCR analysis, 10 μl of 2x QuantiTect SYBR Green PCR Master Mix, 2 μl of the RT product, 0.8 μl of 5 μM Bulge-Loop piRNA forward primer, and 0.8 μl of 5 μM Bulge-Loop piRNA reverse primer were added to each reaction. The volume was adjusted with RNA-free H_2_O. The reaction was incubated at 95 °C for 10 min, followed by 40 cycles at 95 °C for 2 s, 60 °C for 20 s, and 70 °C for 10 s. All reactions were performed in triplicate. U6 was selected as a reference gene according to a previous study [53, 54]. PiRNA expression was normalized to the mean of the reference gene. The relative expression levels of piRNAs were determined using the 2^−ΔΔCt^ method.

### Immunohistochemistry and BRAF ^V600E^ mutation detection

FFPE tissue blocks of 4 μm were deparaffinized and dehydrated. Following routine rehydration, antigen retrieval, and blocking procedures, the sections were incubated overnight with 1:25 dilution of HBME1 antibody (ab2383; Abcam, Cambridge, UK) at 4 °C. The slides were incubated with HRP polymer for 30 min and with hematoxylin as a counterstain for 5 min at room temperature. The sections were analyzed and positive results were recorded for stained cancer cells. The scoring scale used for stained tumor cells was as follows: negative (0), yellowish (1-4), light brown (5-8), and dark brown (9-12). A positive control of PTC was used in each run with the primary antibody being replaced by buffer in the negative controls. HBME1 staining was found in both the cytoplasm and membranous tissue.

Total DNA was isolated from FFPE PTC tissues using the QIAamp DNA FFPE Tissue Kit (catalog #56404, Qiagen, Germany) in strict accordance with the manufacturer’s instructions (AmoyDx BRAFV600E Mutation Detection Kit, Amoy Diagnostics, China, ADx-ARMS). The reaction mixture and positive control from the kit was thawed at room temperature, vortexed for 15 seconds, then centrifuged for 15 seconds. For each sample, 0.4 μl Taq enzyme was added and mixed by vortexing. All samples were centrifuged for 15 seconds and 45 μl of the samples was dispensed into PCR tubes. For each sample, 5 μl of ultrapure water was added and the PCR reaction tube was covered. The reaction parameters were: incubation at 95 °C for 5 min, 15 cycles at 95 °C for 25 s, 64 °C for 20 s, 72 °C for 20 s, 31 cycles of 93 °C for 25 s, 60 °C for 35 s, then 72 °C for 20 s. FAM and HEX (or VIC) signals were collected during the third phase at 60 °C.

### Identification of key piRNAs potential downstream mechanisms

The RNAhybrid algorithm was used to predict the potential target genes of key piRNAs (P < 0.01, binding free energy < 25 kcal). To reveal the potential regulatory mechanism of piRNA, the intersections between the target genes of key piRNAs and DEGs from PTC and normal solid tissues in the TCGA-THCA database [log Fold Change (FC) > 1, False Discovery Rate (FDR) < 0.05] were used to perform functional enrichment analysis.

### Statistical analysis

The CLC genomics workbench 6.0 software was used to map high-quality sequences to the piRNA database with no base mismatch allowed in the alignment process. The fragments of piRNAs were counted followed by Transcripts Per Million normalization. Significant differentially expressed piRNAs were identified as those with FDR values above the threshold (Q < 0.01) and under the criteria of Log2FC > 2. The expression levels of all significantly upregulated piRNAs were validated in all PTC and thyroid nodule samples by RT-qPCR. Paired t-tests and non-parametric tests were utilized to evaluate the relationship between all upregulated piRNAs and AJCC stage. Only piRNAs which were significantly differentially expressed and significantly associated with AJCC stage were included in further biomarker analysis. The expression levels of key piRNAs were corrected by demographic data to construct a multivariate model to distinguish malignant from benign nodules. The nomogram predicting the malignancy of PTC was generated based on the multivariate Cox regression model. The sum of values for the expression levels of key piRNAs, the extent of HBME1 staining, and associated demographic data, provided the probability of PTC. The calibration plot and the ROC curve were used to estimate the calibration and discrimination of the nomogram, respectively.

Only two-sided P value < 0.05 was considered as statistically significant. All of the statistical analyses were performed with R version 3.6.1 software (Institute for Statistics and Mathematics, Vienna, Austria; https://www.r-project.org).

### Ethical approval

This study was approved by the Clinical Research Ethics Committee of Shanghai Tenth People’s Hospital and written informed consents were obtained from all participants for conducting the experiments.

## Supplementary Material

Supplementary Figures

Supplementary Materials

Supplementary Material 1

Supplementary Material 2
