# Supplementary material for: PIWI-interacting RNAs piR-13643 and piR-21238 are promising diagnostic biomarkers of papillary thyroid carcinoma
Source: Aging (Albany NY). 2020 May 19;12(10):9292–310. doi: 10.18632/aging.103206 (PMC7288952; doi:10.18632/aging.103206)
Supplement: Supplementary Figures [file aging-12-103206-s003..pdf]

SUPPLEMENTARY FIGURES

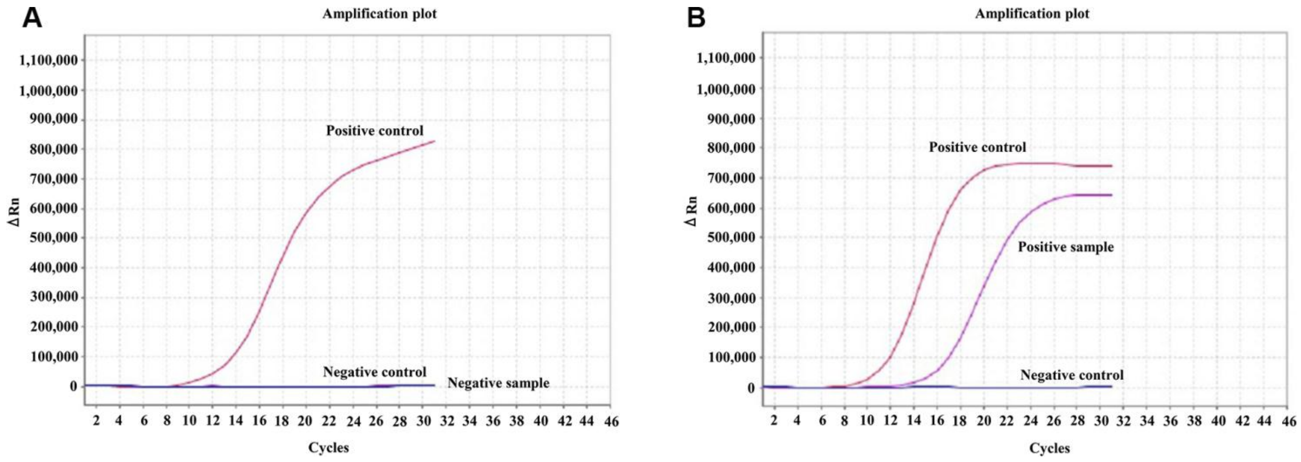

Supplementary Figure 1. Identification of BRAF mutations. (**A**) Sample curve with the wild-type BRAF gene. (**B**) Sample curve with the mutant BRAF gene.

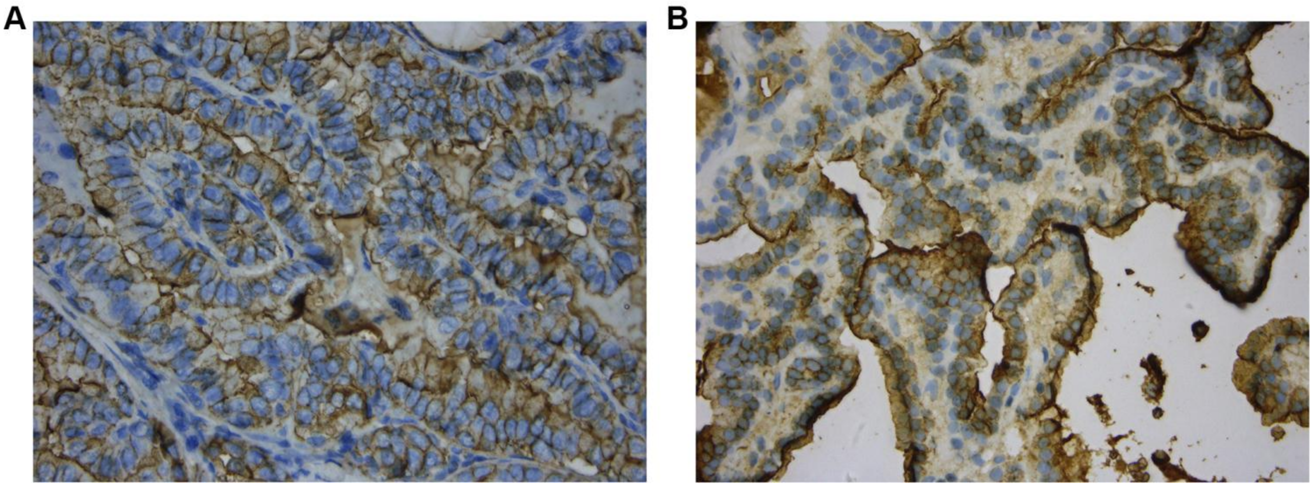

Supplementary Figure 2. In some patients, we also found some ambiguous staining of HBME1, which were concentrated in the benign nodules and non-staining in the malignant nodules (**A**) Diffusely positive HBME-1 cytoplasmic with membranous staining and luminal accentuation is shown in a patient with papillary thyroid carcinoma (X100). (**B**) Diffusely positive HBME-1 cytoplasmic with membranous staining and luminal accentuation is shown in thyroid nodule tissues (X100).
