# Supplementary material for: PIWI-interacting RNAs piR-13643 and piR-21238 are promising diagnostic biomarkers of papillary thyroid carcinoma
Source: Aging (Albany NY). 2020 May 19;12(10):9292–310. doi: 10.18632/aging.103206 (PMC7288952; doi:10.18632/aging.103206)
Supplement: Supplementary Materials [file aging-12-103206-s002..pdf]

SUPPLEMENTARY MATERIALS

Please browse Full Text version to see the data of Supplementary Materials 1 to 2.

Supplementary Material 1. Gene expression profile of 5 thyroid cancer samples by small RNA sequencing (Raw count).

Supplementary Material 2. The binding sites of piR-13643 and piR-21238 to the target genes.
